# Using mobile phone data to map evacuation and displacement: a case study of the central Italy earthquake

**DOI:** 10.1038/s41598-023-48130-4

**Published:** 2023-12-14

**Authors:** Francesca Giardini, Natalia Selini Hadjidimitriou, Marco Mamei, Giordano Bastardi, Nico Codeluppi, Francesca Pancotto

**Affiliations:** 1 Department of Sociology, University of Groningen, Grote Rozenstraat, 31, 9712 TG Groningen, The Netherlands; 2 Dipartimento di Studi Linguistici e Culturali, Università di Modena e Reggio Emilia, Largo S. Eufemia, 19, 41121 Modena, Italy; 3 Dipartimento di Scienze e Metodi dell’Ingegneria, Università di Modena e Reggio Emilia, Via Amendola, 2 - Pad. Morselli, 42122 Reggio Emilia, Italy; 4 Artificial Intelligence Research and Innovation Center - AIRI, Università di Modena e Reggio Emilia, Via Vivarelli, 10, 41125 Modena, Italy

**Keywords:** Natural hazards, Engineering

## Abstract

Population displacement is one of the most common consequences of disasters, and it can profoundly affect communities and territories. However, gaining an accurate measure of the size of displacement in the days and weeks following a major disaster can be extremely difficult. This study uses aggregated Call Detail Records as an inexpensive and efficient technique to measure post-disaster displacement in four Italian regions affected by repeated earthquakes in 2016–2017. By comparing post-disaster mobile phone count with a forecast computed before the earthquake hit, we can compute an index of change in the presence of mobile phones (MPE). This measure, obtained thanks to advanced analytical techniques, provides a reliable indication of the effect of the earthquake in terms of immediate and medium-term displacement. We test this measure against census data and in combination with other datasets. Looking into available data on economic activities and requests for financial support to rebuild damaged buildings, we can explain MPE and identify significant factors affecting population displacement. It is possible to apply this innovative methodology to other disaster scenarios and use it by policymakers who want to understand the determinants of population displacement.

## Introduction

The United Nations Office for Disaster Risk Reduction (UNDRR) defines a disaster as a “serious disruption of the functioning of a community or a society at any scale due to hazardous events interacting with conditions of exposure, vulnerability and capacity, leading to one or more of the following: human, material, economic and environmental losses and impacts” (UNDRR, n.d.). Quarantelli^1^ (p. 682) explores the defining features of disasters, comprising the time frame (disasters are sudden-onset occasions), their disruptiveness towards collective routines, the adoption of unplanned courses of action to adjust to the disruption, and the danger posed to valued social objects. Quarantelli^1^ (p. 682) also emphasized that disasters represent vulnerability, reflecting “weaknesses in social structures or social systems”^2^ (p. 345). The conception of disasters as a weakness of social systems is firmly grounded in the social sciences tradition^3^. Since the relationships in the social system can cause vulnerability, the definition of disasters originates from the notion of social changes. Similarly, Alexander^4^ (p. 29) stresses the impossibility of defining ”disasters with fixed events but by social constructs, and these are liable to change”. Other authors like^5^ support this perspective and refers to disasters as social events. Disasters result from a combination of hazards with vulnerability and exposure of people and assets. Vulnerability refers to the conditions which increase the susceptibility of individuals, communities and systems to the hazards’ impact. According to Wisner et al.^6^ (p. 11), vulnerability is “...the characteristics of a person or group and their situation that influence their capacity to anticipate, cope with, resist, and recover from the impact of a natural hazard”. Vulnerabilities vary by hazard type as they are contingent on several circumstances and unevenly distributed across individuals, households, communities, and locations^7,8^.

Displacement and vulnerability are closely related. Displacement forces villages, towns, or regions to lose inhabitants who need to find alternative accommodations somewhere else^9^. There is extensive literature on the risks to well-being generated by forced displacement (e.g.^10–13^), but less is known about the increasing vulnerability of displacement that can unfold over different periods, from a few days to decades, and the displaced population can move very close to their original location, but also very far away. There is no univocal concept of displacement because it usually depends on the kind of crisis, disaster or conflict that triggered it, and the term itself is not uncontroversial^14^. Here we will use the definition developed by the UNDRR: Disaster displacement is one of the most common and immediate impacts of disasters. It refers to situations where people are obliged to leave their homes or places of habitual residence as a result of a disaster or in order to avoid the impact of an immediate and foreseeable natural hazard, including the adverse impacts of climate change, or a disaster triggered by human-made factors, such as large-scale industrial accidents. Displacement triggered by conflict is not considered disaster displacement^15^. If the citizens do not quickly return to their original places of residence and are involved in the reconstruction of the material and social environment, the loss of human, economic and social capital^16^ can lead to the abandonment of the area. When individuals, firms and service providers must relocate somewhere else, there are net losses in place of community resilience, with parallel increases in vulnerabilities. Infrastructure resilience, i.e., the extent to which there is a restoration of minimum services and functions in a relatively short amount of time, thus allowing people to return to their place of origin^17^, is a first step towards the restoration of communities. The importance of involving citizens and communities in the reconstruction process after the initial evacuation is clearly visible in the case of the L’Aquila earthquake. Imperiale and Vanclay^18^ show how the exclusion of local communities and the top-down planning approach chosen for the reconstruction led to more than 10.000 people still in temporary accommodations 10 years after the earthquake, with many citizens leaving the city and never going back. When population leaves the affected area in a permanent way, this contributes to a decrease in the capacity to cope with additional shocks^16,19^. A vulnerable community can suffer from changes in population in response to direct and indirect impacts, and these variations are hardly stable over time. Usually, variations in population size in a given area are different between the aftermath of the disaster (short-term changes), some months later (mid-term changes), and in the long term, when the reconstruction phase has started because the emergency is over (in this study we are not considering permanent relocation, which might be necessary in some cases). This change is seldom visible in official census data because individuals can autonomously find accommodations without informing the authorities when they flee the disaster area. In other cases, the devastation is so profound that the census records are difficult to acquire and update. In this situation, it is fundamental to have an accurate picture of the number of displaced people and where they have relocated. If a system exhibits the capacity to ‘return to equilibrium after a displacement’^20^, then that system is considered resilient. Displacement is relevant in two of the four priorities outlined in the Sendai Framework for Disaster Risk Reduction 2015-2030 (Sendai Framework), the first agreement of the post-2015 development agenda that provides Member States with concrete actions to protect from the risk of disaster. This study responds to objective (h) in Priority 4 (Enhancing disaster preparedness for effective response and to “Build Back Better” in recovery, rehabilitation and reconstruction), which advocates disaster preparedness and the establishment of rapid and effective responses to disasters and related displacement.

More specifically, this study combines engineering methods to estimate post-disaster displacement using mobile phone data, with a social sciences perspectives on economic factors and community resilience. There are two reasons for this approach.

1. While there exist alternative approaches to estimate displacement, i.e., using aerial images^21^, night-light satellite images^22^, social network, crowd-sourced and damage assessment data^23–25^, novel data sources can integrate existing information and provide further insights. Mobile phone data (often called Call Detail Records - CDRs) is a natural candidate in this regard as it combines ready available data with an almost complete population coverage.
2. Information about CDRs (short-term) can then be integrated with other elements, for instance information about the economic structure of the area or about the reconstruction process (mid-term). Understanding how factors other than the sheer magnitude of the disaster might be contributing to displacement is essential for the design of policies that can effectively counteract population displacement and can improve community resilience.

Thanks to this novel combination of data and methods, we aim to answer the following two research questions:

1. *How to quantify population displacement from fully aggregated mobile-phone data?* Most of the approaches reported in the literature^26–31^ derive displacement information directly from the data (i.e., looking at where each mobile phone moves after the disaster). Using fully-aggregated data, this kind of approach is unusable as privacy regulations do not allow to track phones but only to measure their presence in an area (see Section “Methods”). Therefore, in this study we had to develop a novel approach to quantify displacements. While we applied our method to a specific case study - the earthquake that struck Central Italy in 2016- the approach discussed in this study is more general, and can be implemented it in other emergencies once data are available—see Section “Methods”.
2. *What is the relationship between population displacement, economic activities and infrastructural damages in the selected case study?* After measuring displacement resulting from the Central Italy earthquake, it is interesting to understand the main factors impacting it. Proximity to the earthquake epicentre and the size of damage suffered naturally have a high impact, but there are other important factors. In particular, we focus on the geographical and economic characteristics of the affected areas. Because disasters affect various economic activities in a heterogeneous way, population changes are heterogeneous across municipalities. In our case study, manufacturing or logistics, for instance, require physical infrastructures that are easily damaged by an earthquake. Moreover, these industries present more complex inter-dependencies among production plants and supply chains, all features heavily disrupted by a disaster. Therefore, municipalities with an high number of such companies are likely to suffer more displacement because of the destruction.

In this study, we focus on CDRs (Call Detail Records), which offer a quick way to obtain data about post-disaster mobility. CDRs are automatically and routinely collected by telecom operators and contain approximate information about the location of every mobile phone connected to their network^32–36^. It is relevant to notice that while the use of mobile phone data at the *individual*-level (i.e., tracking individual mobile phones) is very much constrained due to privacy reasons, the use of *aggregated and fully anonymized* mobile phone data is easily accessible. Most telecommunication operators have commercial offers to provide aggregated and anonymous data about people’s presence and mobility. The main idea is that mobile phones precisely localize a substantial fraction of the population with minimal cost and intervention. Unlike census data or data collected by municipalities at selected time points, CDRs provide a continuous estimate of the situation, allowing researchers to identify changes in relocation patterns and displacement as they happen.

In the literature there are many examples on the use of CDR data in the context of disaster risk reduction. Counter-intuitively, the vast majority of the works presented in the literature focus on individual-level data (that is hard to acquire), while they often disregard aggregated-level data.

Many scholars have focused on describing mobility patterns to understand post-disaster population displacement at different periods in time. In^28^, mobile phone data allowed for predicting people’s mobility during a disaster. The authors analysed data about mobile phones 42 days before and 341 days after the Haiti earthquake. Their results show that the earthquake caused an increase in population movement, an increase in daily distances in the affected areas and more heterogeneity in terms of average travel distance. They also found that people’s movement was more predictable after the disaster because of the high correlation with the places where people had strong social relationships. Similarly, in another work on the Haiti earthquake^29^, authors found that 20% of the population emigrate from Port-au-Prince during the first 19 days after the earthquake. To validate the results, the authors compared with the estimates of the National Civil Protection Agency based on the number of ships and buses leaving the area and the survey performed by the United Nations about migration after the earthquake. Thanks to mobile phone data, the authors found the regions where most of the population migrated, thus offering support to policymakers for planning and relief efforts. Similar analysis performed in the context of the Nepal earthquake in 2015^37^, the landslide in Japan in 2014^26^, or the Mozambique cyclone in 2019^38^ show the evolution of population mobility patterns after the disasters and the return patterns to affected areas in the following days and weeks. In a recent study on mobile phone data published by^31^, the authors suggest the importance of inter-city connectivity in post-disaster recovery. Using mobile phone call data records, the authors show that the cities with high inflows and outflows of people before the disaster were more resilient and recovered faster.

Some recent works started comparative analysis among multiple disasters analyzing pre- and post-disaster individual mobile phone trajectories to test the generality of this approach^39,40^. In^27^, mobile phone data enabled the study of the population recovery patterns after extreme events. The authors found very similar behaviours in five different locations across world. They explained the differences using income, population, housing damage rate and connectivity with other cities. When they analysed the recovery time distribution, they found that most people returned to their homes within two weeks. Furthermore, they tried to explain the displacement rate based on socioeconomic variables, infrastructure recovery and accessibility. The results showed that, at different times, the infrastructure recovery became more relevant compared to the house’s damage rates.

The works in^41,42^ analyze *aggregated* mobile phone data in the context of the earthquake that shook Ecuador on April 2016. The analyzed data contains information about the *home area* of the monitored phones, which allows estimating displacements more easily. Similarly, the work in^43^ is one of the few dealing with aggregated mobile phone data. They identify anomalies in the data collected during multiple events affecting some US states in 2018 (e.g., hurricanes Florence and Michael).

Mobile phone data can indirectly measure the time needed for a given area to recover. The Resilience to Emergencies and Disaster Index proposed by^44^ assesses the recovery period of neighbourhoods impacted by a storm to prioritize limited resource use. The authors considered mobile phone data as a proxy of neighbourhood activity, social infrastructure, community connectivity, physical infrastructure, economic strength and environmental conditions as distinct variables and computed the indicator as the normalized weighted sum of these variables. According to^45^, mobile phone data alone does not explain the motivation why people move and relocate in a certain way after extreme events. Thus, it is necessary to perform data fusion on different datasets or to combine big data with smaller datasets, such as surveys. In this regard^30^, analysed migration patterns based on mobile phone data and the national survey that included questions on temporary and seasonal migration. Furthermore, the authors underline the importance of considering short-term and long-term displaced people in the definition of migration. Their results evidence that temporary migration can be detected using mobile phone data.

Our research contributes to this consolidated body of work by providing new insights into data and methods. More specifically:

1. Unlike most papers discussed so far, we do not use data that can be associated with a single mobile phone because this would posit several limitations in terms of privacy and would hinder the actual applicability and reproducibility of our method and analysis. Our approach allows us to overcome this limitation by using anonymous and aggregated Call Detail Records (CDRs) that telecom companies routinely collect and sell for business/commercial purposes. For this reason, we acquired data from a wide area comprising four regions, 663 municipalities and spanning three years – such a large extent is uncommon in the literature. Such aggregated data required us to develop a novel method to measure population displacement based on non-autoregressive time-series forecasting. Although such a forecasting approach is not novel per-se, the application to mobile phone data is new (see Section “Methods”).
2. In this study, we combine CDRs data with data about economic activities, companies’ information and government funding for reconstruction. Using a large dataset on commercial and industrial companies of any size and sector, we introduce in the model very detailed and relevant information on the kind of economic activities present in a given municipality. Information about the industrial composition at the municipality level is valuable to embed the effects of the disaster into the existing economic setting. As an additional step, we link CDRs with data about the funding requests for the reconstruction of residential, commercial and industrial buildings to understand damages and mid-term trends in people’s returning home. These datasets offer a multifaceted picture of post-disaster damage and displacement and suggest relevant directions for policymakers.

## The case study

What is known as the ‘Central Italy earthquake’ is a sequence of different shocks happening over a period of five months in 2016^46^. The first earthquake hit at 3.36 a.m. on August 24, 2016, with a 5.9 magnitude. The affected area, administratively divided under four Italian regions, consists of Abruzzo, Lazio, Marche and Umbria. During three days, above 2000 aftershocks were recorded following the main seismic event, extending along 25 kilometres through Amatrice and Norcia. The largest aftershock occurred near Norcia (5.3 Mw) at 04:33 on August 24, 2016. The Italian Civil Protection reported that 299 people died. On September 5, 2016, the number of people needing assistance was 4807. On October 26 and 30, new violent shocks rock the same area, especially between Umbria and Marche, already markedly affected by the August 24 earthquake. Specifically, the October 26 event consisted of two 5.4 and 5.9 magnitude earthquakes. On October 30, a new shake caused the collapse of buildings^47^. On January 18, four earthquakes of magnitude higher than 5 hit the area, and Lazio and Abruzzo’s regions were the most affected. In particular, a 5.1 event was registered at 10:25, followed by other earthquakes: the second at 11:14 (5.5 Richter magnitude), the third at 11:25 (5.4 Richter) and the fourth at 14:33 (5 Richter). The affected territory is mountainous, with more than 70 per cent of the surface located at an altitude higher than 900 metres. This mountainous configuration means that there is an uneven distribution of urban areas: the majority of the inhabitants live in several small centres surrounded by widespread rural zones within a mountainous landscape, most of which are isolated and accessible only by minor roads^48^. According to Decree-Law 17 October 2016, n. 189, 140 municipalities that fall into the “seismic crater” are entitled to government funding for private and public reconstruction. Overall, this sequence of earthquakes heavily affected the whole area, although the 140 municipalities close to the earthquakes’ epicentres—referred to as the *seismic crater,* were the most damaged. According to governmental reports^49^, the total population living there before August 2016 was 581.885. Concerning the estimate of the costs of the Central Italy earthquakes, the Italian Government provided them in June 2022. The estimated request for funding at the time was of 22.695 units, corresponding to a value of 7.669.353.627 euros. The expected amount of further requests is 27.350 for 11.794.161.941 euros, reaching 50.045 units in total that need reconstruction^49^.

## Results

Our analytical strategy consists of the following steps:

1. Measuring the impact of the earthquake in the number of mobile phones present in the area. The objective is to evaluate the relationship between this variation and the people’s presence in the area in the short-term. We propose the Mean Prediction Error (MPE) to compute the displacement in population, and assess how strongly this correlates with the observed changes in population according to census data.
2. Analyzing the relationship between MPE and other important variables, namely reported damages, distance from the epicentre, structural characteristics, and economic activities, to explain variations in population displacement in the mid-term.

### Monitoring population via CDRs

**Figure 1:**
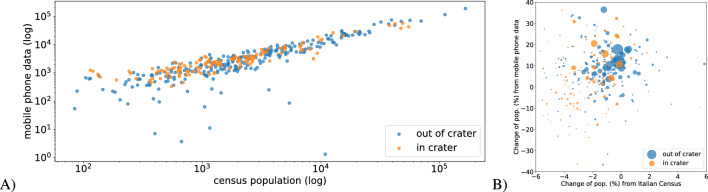
Each dot represents a municipality. Blue dots are outside the earthquake crater area, and orange dots are inside the crater area. (**A**) People count from the Italian census (ISTAT) and mobile phone data. (**B**) Population change from the Italian census (ISTAT) and mobile phone data, the size of dots represent the municipality population.

In the first set of experiments, we use mobile phone data to quantify people’s displacement after the earthquake. Figure 1A compares the number of residents in the municipalities of the area under study with the corresponding data from mobile phones (computed as the average number of phones at night – see Section “Methods”). There is a clear linear relationship among the two measures ($\beta = 1.17\;(0.01)$, $R^2=0.95$), confirming the representativeness of the mobile data. Figure 1B compares the percentage change in residents’ population as measured by official statistics between 2017 and 2016 and the change in residents’ population as measured by mobile phone data. In particular, we calculate the number of resident changes as measured by official statistics (OCHG—Official CHanGe) as follows:

$$\begin{aligned} OCHG = \frac{\text {n.residents end 2017} - \text {n.residents beginning 2016}}{\text {n.residents beginning 2016}} \end{aligned}$$

Similarly, we calculate the change in residents’ population measured by mobile phone data (PCHG—Phone CHanGe) as:

$$\begin{aligned} PCHG = \frac{\text {avg. n. phone at night after earthquake} - \text {avg. n. phone at night before earthquake}}{\text {avg. n. phone at night before the earthquake}} \end{aligned}$$

There is almost no relationship between the two measures. Looking at the small range of variation in official statistics and considering the destruction of some municipalities by the earthquake, we can safely assume that official statistics do not provide an accurate population dynamics estimate. In these extreme situations, people do not promptly communicate their change in residency. However, in the context of earthquakes and natural hazards, a timely representation of people displacement is critical to organize relief activities and prioritizing resources. Therefore our results show the importance of using CDRs *per-se* in a more precise manner for monitoring population dynamics.

**Figure 2:**
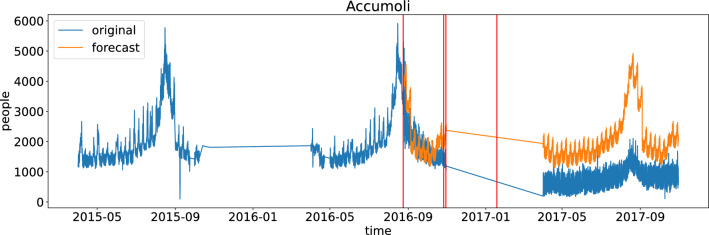
Mobile phone data was collected in the Accumoli municipality (blue). Forecast data based on the pattern exhibited before the earthquakes (orange). Red vertical lines are associated with the four main earthquakes that hit the region. Actual data is 50% lower than forecast ones.

To understand the impact of the earthquake in the area, we trained a non-autoregressive forecasting model to predict population dynamics as measured by mobile phones (see Section “Methods”). We trained the model on individual municipalities with data collected before the first earthquake hit the region (August 24, 2016). Then, we compared the prediction results with actual data, using ‘testing’ data after the first earthquake hit. Figure 2 illustrates the results in Accumoli, one of the municipalities hit the hardest by the earthquake. It is possible to see that forecast (orange) is much higher than the actual data (blue). The idea of this approach is that, since the forecasting algorithm receives only data *before* the earthquake happened, the forecast results (orange line) can be interpreted as the counterfactual scenario as if the earthquake had not occurred. In the “Discussion” Section, we discuss the limitations of this idea. The main advantage of MPE over PCHG is that MPE automatically deals with data seasonality.

Following this approach, for each municipality, we compute the mean (percentage) prediction error (MPE) as:

$$\begin{aligned} MPE = \frac{1}{N}\sum _t^N\frac{\text {actual}_t - \text {forecast}_t}{\text {forecast}_t}. \end{aligned}$$

In this way, if forecast values are higher than actual ones, the *MPE* is negative and represents a percentage decrease in the population. Since there were multiple earthquakes spanning approximately six months, we further decompose MPE in two time-bound measures.

We compute $MPE\;INIT$ focusing on ‘testing’ data from 25 August 2016 to 30 October 2016. This time frame coincides with the period between the aftermath of the initial shake (24 August 2016) and the two months after it. We also compute $MPE\;END$ focusing on ‘testing’ data from 1 April 2017 to 30 October 2017. In this period, the medium-term effects of the earthquake become visible, allowing for an interesting comparison between the two-time frames. We chose these time frames to split between $MPE\;INIT$ and $MPE\;END$ according to data availability.

**Figure 3:**
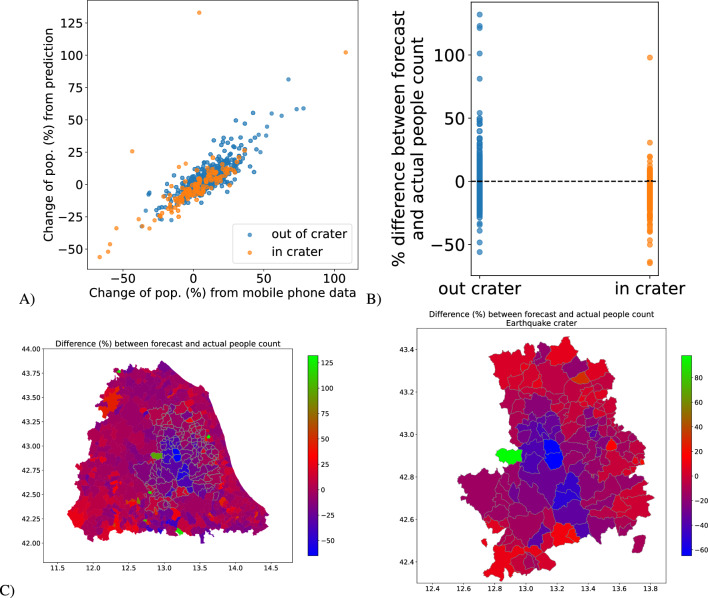
Each dot represents a municipality. Blue dots are municipalities outside the earthquake crater area, and orange dots are those inside the crater area. (**A**) Relationship between PCHG and MPE. (**B**) MPE for all municipalities under study. (**C**) Map, showing MPE for the Central Italy municipalities (left) and zoom in on the crater (right). Maps have been drawn using python libraries: *matplotlib 3.5.2* and *geopandas 0.2.12* – https://www.anaconda.com.

Figure 3A shows the relationship between MPE and PCHG and the good linear relationship between the two variables($\beta = 0.72\;(0.02)$, $R^2=0.64$). Figure 3B shows MPE results for all the municipalities. In this case, the variation in mobile phone data proves accurate. The area closer to the epicentre and mostly affected (the so called *seismic crater*) has the highest negative MPEs indicating that the municipalities had the highest decrease in population.

Similarly, Figure 3C shows a map of central Italy (left) and focuses on the earthquake crater area (right). It is possible to see that, in general, the crater area had the most significant reduction in population size, measured as the highest negative MPEs. However, there is high variability, and some municipalities in the seismic area witnessed an increase in their population.

### Population change, economic activities and damage impact

In this second set of experiments, we create linear models to explain the variation of MPE in terms of geographic and economic variables. Given the variability in MPE (and PCGH) observed in Fig. 3, we created different models to explore the origin of this variability. Our intuition is that other than the earthquake magnitude and associated damages, the kind of economic activities in each municipality might impact population dynamics. For example, since earthquake usually affects built infrastructures, agricultural activities are likely to be less affected than manufacturing or logistics, but also because the latter typically involves more complex inter-dependencies among production plants, supply chains and logistics.

The following analysis provides evidence about this link between economic activities and displacement in municipalities. We developed three models (linear regression) to explain changes in MPE in the affected areas.

1. The model in Table 1 uses *geographic features* and *earthquake damage features* as covariates (independent variables). *Geographic features* of the municipality are the distance from the epicentre and median altitude (ALT MED). The epicentre distance can be a simple proxy of the effects of the earthquake on a given area (of course, there are many more factors determining the consequences of the earthquake). In addition, given that the seismic area comprises several small centres surrounded by widespread rural and mountainous zones, most of which are isolated and accessible only by minor roads, we considered the municipality’s (median) altitude as a simple proxy for municipality isolation. *Earthquake damage features* are the number of requests for rebuilding (RCR) from private (RCR PRIV) and public (RCR PUB) entities that are a proxy for earthquake destruction (see Section “Methods”).
2. The model in Table 2 uses *geographic features* and *economic sectors’ features*. The considered classification of economic sectors is the ATECO (1-letter code) which contains unique identification codes for every economic activity. To limit the number of variables, we consider only those sectors contributing to at least 1% of the total number of companies and 1% of the total revenue of the area (see Section “Methods”, also for comparison with the results with all the sectors considered).
3. The last model (Table 3) combines all the previous covariates: *geographic features*, *earthquake damage features* and *economic sectors’ features*.

In Table 1, we see a positive relationship between the distance from the epicentre and MPE in all three measures, indicating the accuracy of MPE in capturing the displacement of inhabitants, both in the first response to the event as well as a later one. The number of requests for rebuilding from the public sector (RCR PUB) is significant and negative: the higher the amount of RCR, the lower the MPE (greater negative MPE, e.g., − 50% in cell phone presence), meaning that there is a higher number of displaced people, probably because of the destruction. The number of requests for rebuilding from the private sector is significant (and negative) only for MPE INIT. RCR PRIV is a proxy for the damage to homes and private buildings. This damage implies the immediate displacement of people. Therefore, MPE INIT better captures displacement, while RCR PUB represents public buildings and infrastructures. Finally, MPE END represents the impact on the long-term habitability of the area. In this analysis, we considered the number of requests presented for funding. We conducted a similar study using the initial assessment of the damage of the total amount in Euros of the requests obtaining similar results (see Section “Methods”).

In Table 2, we added the industrial sectors to explore what might affect MPE (a short description of the variables associated with industrial sectors in the regression is in Table 4). First, the distance from the epicentre positively affects MPE in all three measures. The altitude of the municipality is significant for MPE END and MPE, indicating that locations with higher altitudes (likely to be more rural and less connected) have a higher decrease in population. Concerning economic sectors, we observe that constructions, hospitality and manufacturing affect MPE INIT. The effect is present, also in MPE END for logistics, manufacturing, whole retail sales and finance, but not for hospitality and construction. These two sectors show no significant impact on MPE END. This effect may be because community resilience does not relate to hospitality and construction. However, these activities relate to the immediate responses to the disaster, such as rescue operations and activities to secure crumbling buildings and houses. Therefore, they can drive an increase in the number of people in the area. The evidence presented here suggests that more modern economic sectors (secondary and tertiary), which require supply chains, logistics and interconnections with other sectors and geographical areas, have been heavily affected by the disaster, and this effect is long-lasting. Furthermore, this suggests the need for more active policy interventions to support the recovery of these industries and economic sectors.

**Figure 4:**
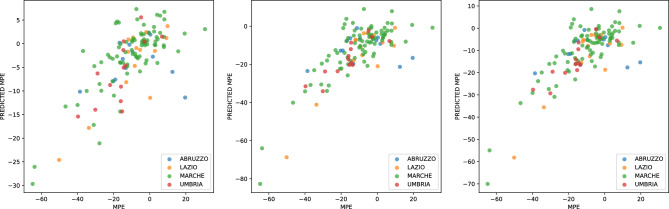
Actual and regressed MPE, MPE INIT and MPE END in the earthquake crater zone using the model with covariates: *geographic features*, *earthquake damage features* and *economic sectors’ features*.

Finally, the coefficients in Table 3 support the previous conclusions. With more variables, the model has a greater explanatory power ($R^2$). Figure 4 shows the model result with the actual MPE, MPE INIT and MPE END and the predicted ones. The reported graph illustrates the fitting of the models. It is worth explaining that the model is fitted on 123 municipalities (observations) of the seismic crater, while official reports identified 140 in that area. This mismatch is due to missing data for 17 (12%) municipalities. We did not consider these municipalities in the model.

**Table 1:** Model to explain MPE (MPE INIT, MPE END, MPE) in terms of geographic and damage features.

	MPE INIT	MPE END	MPE
Distance from epicenter	0.308***	0.602***	0.532***
	(0.083)	(0.168)	(0.134)
ALT MED	0.001	0.001	0.001
	(0.003)	(0.005)	(0.004)
RCR PRIV	$-$20.150*	$-$10.629	$-$12.904
	(11.831)	(23.369)	(18.611)
RCR PUB	$-$110.132**	$-$369.151***	$-$307.251***
	(48.978)	(99.943)	(79.503)
Observations	123	123	123
$R^2$	0.467	0.472	0.520
Adjusted $R^2$	0.449	0.454	0.504
Residual Std. Error	6.753	13.781	10.962
F Statistic	25.899***	26.349***	31.948***

*p < 0.1; **p < 0.05; ***p < 0.01.

**Table 2:** Model to explain MPE (MPE INIT, MPE END, MPE) in terms of geographic and economic features.

	MPE INIT	MPE END	MPE
Distance from epicenter	0.437***	0.833***	0.739***
	(0.078)	(0.158)	(0.127)
ALT MED	$-$0.002	$-$0.012**	$-$0.010**
	(0.003)	(0.006)	(0.005)
Agricolture	$-$5.893	$-$8.158	$-$7.617
	(4.888)	(9.838)	(7.942)
Real estate	2.358	$-$18.597	$-$13.590
	(9.329)	(18.777)	(15.158)
Hospitality	$-$11.096*	$-$4.085	$-$5.760
	(6.271)	(12.621)	(10.189)
Manufacturing	$-$6.869*	$-$18.696**	$-$15.870***
	(3.546)	(7.138)	(5.762)
Tech services	$-$7.535	$-$26.066	$-$21.638
	(11.358)	(22.860)	(18.454)
Whole/retail sales	$-$4.785	$-$14.255*	$-$11.992*
	(3.998)	(8.047)	(6.496)
Constructions	$-$8.232**	$-$5.361	$-$6.047
	(3.801)	(7.650)	(6.176)
Business support services	$-$3.342	$-$6.245	$-$5.551
	(22.615)	(45.518)	(36.744)
Communication and info serv.	19.008	$-$46.248	$-$30.653
	(49.252)	(99.131)	(80.024)
Logistics	$-$12.423	$-$46.660**	$-$38.478**
	(10.323)	(20.776)	(16.772)
Healthcare	$-$28.748	$-$25.464	$-$26.249
	(33.740)	(67.910)	(54.820)
Finance	$-$2.998	$-$38.056**	$-$29.678**
	(7.261)	(14.614)	(11.797)
Utilities	$-$9.577	$-$31.936	$-$26.593
	(10.862)	(21.863)	(17.649)
Observations	123	123	123
$R^2$	0.467	0.485	0.518
Adjusted $R^2$	0.392	0.413	0.451
Residual Std. Error	7.098	14.287	11.533
F Statistic	6.239***	6.723***	7.671***

*p < 0.1; **p < 0.05; ***p < 0.01.

**Table 3:** Model to explain MPE (MPE INIT, MPE END, MPE) in terms of geographic, damage and economic features.

	MPE INIT	MPE END	MPE
Distance from epicenter	0.274***	0.560***	0.492***
	(0.086)	(0.171)	(0.136)
ALT MED	0.001	$-$0.004	$-$0.003
	(0.003)	(0.006)	(0.005)
RCR PRIV	$-$20.711*	$-$11.338	$-$13.578
	(11.799)	(23.418)	(18.624)
RCR PUB	$-$153.953***	$-$420.597***	$-$356.876***
	(51.343)	(101.904)	(81.041)
Agricolture	$-$5.824	$-$10.518	$-$9.396
	(4.653)	(9.236)	(7.345)
Real estate	1.440	$-$20.759	$-$15.454
	(8.734)	(17.335)	(13.786)
Hospitality	$-$14.725**	$-$10.620	$-$11.601
	(5.942)	(11.793)	(9.379)
Manufacturing	$-$8.457**	$-$22.601***	$-$19.221***
	(3.348)	(6.645)	(5.285)
Tech services	$-$10.207	$-$33.978	$-$28.297*
	(10.681)	(21.198)	(16.858)
Whole/retail sales	$-$7.815**	$-$21.728***	$-$18.403***
	(3.836)	(7.613)	(6.054)
Constructions	$-$11.890***	$-$14.706**	$-$14.033**
	(3.717)	(7.378)	(5.867)
Business support services	$-$9.999	$-$23.426	$-$20.218
	(21.258)	(42.192)	(33.554)
Communication and info serv.	12.009	$-$52.880	$-$37.373
	(46.200)	(91.695)	(72.923)
Logistics	$-$9.130	$-$40.085**	$-$32.687**
	(9.693)	(19.239)	(15.300)
Healthcare	$-$41.674	$-$52.623	$-$50.006
	(31.730)	(62.976)	(50.083)
Finance	$-$5.110	$-$45.172***	$-$35.598***
	(6.880)	(13.654)	(10.859)
Utilities	$-$12.181	$-$38.209*	$-$31.989**
	(10.190)	(20.224)	(16.083)
Observations	123	123	123
$R^2$	0.542	0.570	0.609
Adjusted $R^2$	0.467	0.500	0.546
Residual Std. error	6.643	13.184	10.485
F statistic	7.296***	8.180***	9.627***

*p < 0.1; **p < 0.05; ***p < 0.01.

## Discussion

When sizeable portions of the population move out because of a disaster, massive economic and social losses are observed in the affected areas. Being able to quickly and reliably measure these changes inexpensively and unobtrusively is very important. This study shows that aggregated mobile phone data can represent a reliable indication of population variation in the aftermath of an earthquake. Moreover, the combination of CDR with the census, economic and administrative data from four Italian regions affected by repeated earthquakes in 2016 allows us to identify key characteristics that can help to explain displacement dynamics. Our results show that analyzing CDRs can be extremely useful in the aftermath of the disaster, but also that they can be fruitfully related to other data sources related to the reconstruction process.

Mobile phone data (CDRs) can naturally enrich and complement other approaches to estimate displacement (e.g. aerial images^21^, night-lights satellite images^22^, social network, crowd-sourced and damage assessment data^23–25^) offering ready availability, extremely long monitoring periods, and almost complete coverage of the population. Differently than mobile phone data *at the individual level*, which are very hard to get due to privacy regulations, *aggregated* data are readily available without privacy concerns. The method proposed here for the analysis of CDRs data offers the opportunity of knowing soon after the disaster (the time frame depends on data availability) how many people moved out of the affected area (measured by the introduced MPE value).

The approach used to compute this displacement is one of the main novelties of our work. We calculate the value MPE as the mean percentage prediction error between the actual data and a non-autoregressive forecasting model trained with only data collected before the earthquake hit. As the model never receives data during or after the earthquake, we interpret its forecast as the counterfactual scenario as if the earthquake had not happened^50^. We do not claim this to be a full-fledged causal analysis, and we are aware that the results might be partial: there are likely some confounding factors not accounted for by our model. Nonetheless, we are confident that our analysis captures, at least, part of the counterfactual scenario.

This displacement estimate based on mobile phone data can be extremely useful in emergency intervention planning, especially in remote or widely spread areas. In the initial emergency stage, knowing how many people need alternative accommodations, how many should receive which forms of economic support and where there is a high expectation of population change are all relevant variables that can stir the emergency management phase in different directions.

Third, our study shows how fruitful it is to combine CDRs data with existing datasets to explore which characteristics of the disaster and the area can explain variations in the number of residents. In our study, the calculation of MPE and its combination with data about the economic activities in the affected areas shows that some areas are more vulnerable than others, and further studies would help understand better the extent of these vulnerabilities. The need to identify vulnerable areas is especially relevant in a country like Italy that, because of the particular morphology and geophysical context of its territory, is one of the five European countries with the highest probability of a disaster and related economic loss^51^. Readily available information about the impact of an earthquake in terms of immediate displacement can be part of strategic planning for temporary housing that identifies opportunities and constraints for temporary housing before the disaster^52^. Local, regional and national institutions face what has been called the “post-disaster recovery dilemmas”, i.e., the need to balance short-term and long-term needs for vulnerability reduction^52^. There are no obvious solution to such an issue, which greatly varies depending on the situation, but our results can inform policies aimed to support financially and logistically those industries most affected by the disaster, those that are supposedly more relevant for the area’s resilience and the country’s economic growth. Our results are limited in time, location, and kind of disaster, but they can provide a valuable avenue for future research. Even if our methodology needs to be further refined, we point out the relevance of the indications about the relationship between the kind of economic activities and the funding requests. In our data, hospitality is the sector that suffered the least, while the most affected municipalities are those characterized by construction and manufacturing activities. This finding could indicate that, in vulnerable areas, some economic activities could be more resilient to disasters, and their presence can positively affect the reconstruction.

In addition, by using administrative data about the requested funds to rebuild homes and combining it with economic data and MPE, we can offer an overview of the reconstruction process. By calculating MPE, the effective damage and the intention to stay (as the requests for funds necessary to rebuild or repair the damaged houses), we observe that MPE seems to be a good predictor of the latter variable. This finding confirms its efficacy as a medium-term indicator in a situation in which displaced citizens have relocated without registering as residents in another municipality, thus creating difficulties for institutional actors to know the actual magnitude of the displacement and to take adequate measures to assist the population.

For instance, when deciding where to locate aid centres and shelters, it is important to know where most of the population has moved. This data can enrich the vast pool of information (e.g., established emergency plans, conditions of buildings, roads, and transports) to guide and optimize the positioning of such facilities.

A further avenue for future research is using data about the post-earthquake reconstruction process that the Italian government has made available for three different earthquakes that happened in the last 15 years (L’Aquila, Emilia-Romagna and Central Italy). Our results indicate that using data about expected and actual funding requests for rebuilding could offer a valuable indication of displacement and its medium-term consequences. It is worth stressing that physical reconstruction does not necessarily lead to community recovery, and several factors can affect how much communities can “build back better”^18,53^. However, our data and analysis do not allow us to draw any conclusions about community recovery. Qualitative and quantitative individual data, like interviews, focus groups and surveys, would be needed to investigate the relationship between infrastructural reconstruction and community restoration.

As a final remark, we want to point out that in this work, we use data from the Telecom operator in 2015-2017, as the earthquake hit the region during that period. Mobile phone data help analyze displacement right after the disaster and in the months and few years after the fact when official data are often missing or incomplete. Longer-term observations (e.g., using data available now - 6 years after the fact) are less useful as official statistics and data about the reconstruction take the lead role in such a time horizon. Nevertheless, the proposed methods can be applied to other disasters in other years without modifications, and data gathered after different kinds of disasters across diverse countries would be necessary to ground the validity of this approach.

## Conclusions

From the above analysis, it is possible to draw the following conclusions which answer the stated research questions: (i) completely anonymized and aggregated mobile phone data can monitor population displacement after disaster scenarios. The proposed method computes displacement by comparing the actual mobile phone count with the forecast computed before the earthquake hit (see Fig. 3). (ii) Geographic features (epicentre distance and altitude) and damage-related features (public and private RCR) explain part of population displacement (see Table 1), (iii) also the industrial base and composition of the regions contribute to explaining part of population displacement (see Table 2). A better-performing model combines the above features (see Table 3).

## Methods and supplementary analysis

### Data

In this work, we processed four different sources of data to extract and analyze behavioural patterns before and after the earthquake in central Italy in 2016: (i) aggregated Call Detail Records; (ii) economic activity data; (iii) data about damage and recovery activities and (iv) census and demographic data.

On the one hand, aggregated Call Detail Records are the data at the basis of the proposed approach. They allow us to compute population displacement (MPE) at a fine-grained scale. In principle, they are available in quasi-real-time for emergencies and provide timely and valuable information. On the other hand: economic activity, damage, recovery, census and demographic data are functional to the analysis presented in the results to explain the factors affecting MPE.

#### Call detail records

**Figure 5:**
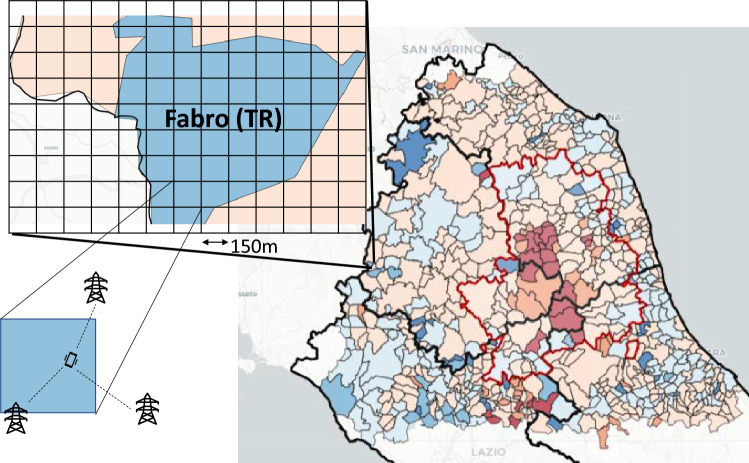
Cell phones are localized by the telecom operator in an area of 150 * 150 meters, called a pixel. Aggregated counts for each pixel is the data provided by the telecom operator in compliance with privacy regulation. Data have been further aggregated at the municipality level to make it more manageable and have areas comparable with other datasets. Maps have been drawn using python libraries: *matplotlib 3.5.2*, *geopandas 0.2.12* and *folium 0.14.0*—https://www.anaconda.com.

We obtained from TIM, the largest mobile phone operator in Italy at the time of the data collected (30.2% market share^54^), a large dataset of *aggregated* CDRs (Call Detail Records) measuring the number of cell phones localized by the network in a given area of the territory. Data covers the whole of Central Italy, divided into a matrix of small square areas of 150 meters side, called pixels. The dataset consists of the number of devices in each pixel every 15 minutes. The localization procedure is proprietary to the telecom operator. Localization is based on signal strength triangulation at the different antennas (see Fig. 5). The data contains only people count. It is not possible to reconstruct the movement of individuals. In addition, to remove the possibility of singling out individuals, the value of any pixel with fewer than N individuals (typically N = 4) is set to zero. As the data measures mobile phones from a single operator, we linearly scale numbers to consider the operators’ market share in the area. Nevertheless, our data do not fully compensate for people not having mobile phones (e.g., the presence of children and elderly). Further research would be needed to address this aspect. However, for the presented results, it is worth considering that: (i) Italy is one of the countries with the higher number of mobile phone operations, (ii) our data strongly correlates with census data—see Fig. 1A- hinting to a limited effect of this bias.

The mobile phone dataset spans the following periods: from 2015-04-01 to 2015-10-30, from 2016-04-01 to 2016-10-30 and from 2017-04-01 to 2017-10-30, to capture presence before and after the earthquake.

To make the dataset more manageable and have areas compared with other datasets, we aggregated data at the municipality level and the 1-hour time interval. This procedure reduces possible biases due to setting small counts to zero, as the aggregation over whole municipalities leads to higher counts.

**Figure 6:**
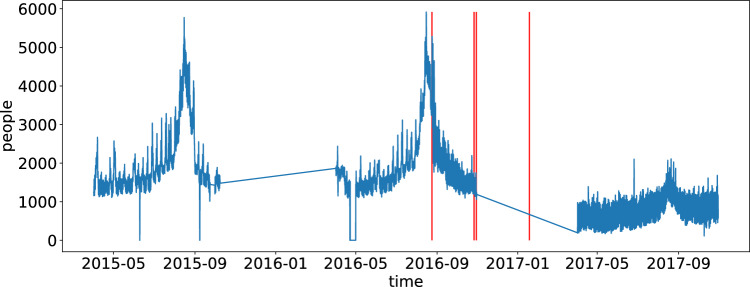
Mobile phone collected for the municipality of Accumoli. The time series records the number of mobile phones at an hourly resolution. Red vertical lines are associated with the four main earthquakes that hit the region.

Overall, we have data for 663 municipalities with 14,420 hourly measures each. Figure 6 shows an example of the data collected for each municipality.

We want to emphasize again that we deployed fully aggregated data. While individual mobile phone traces are very privacy sensitive, as they allow tracking single phones and, therefore, users, aggregated CDRs counting the number of phones in a given area is privacy compliant as the aggregate number does not allow to single out individuals. The operator carried out the entire aggregation process in compliance with privacy regulation (GDPR), and most telecom operators have commercial activities to exploit this kind of data. The possibility to deploy this kind of data is a strength for the actual applicability of our approach. While individual data would require strict scrutiny and control from privacy regulators, telecom operators sell aggregate data. Even more, the value recognition of this data for applications like the one described should push the government and public bodies to create partnerships with a data provider to ensure the prompt availability of this data.

#### Economic activity data

AIDA, created and distributed by Bureau van Dijk S.p.A., is a database, available upon subscription, containing the financial statements and personal and product data of all Italian companies, both active and bankrupt. For each company, the dataset includes the industry/sector, the number of employees and the legal and operating addresses, which allows for localizing them at the municipality level.

Table 4 shows the overall number of companies present in the seismic area.

**Table 4:** Overall composition of companies in the crater area. ATECO 1-letter classification.

ATECO	N	Revenue (K)	Employees (K)	Empl. per Comp.	Description
Whole/retail sales	9852 (18%)	28354 (31%)	66 (15%)	6	Whole and retail sales companies
Manufacturing	9660 (18%)	36901 (40%)	173 (39%)	17	Manufacturing activities
Constructions	9443 (17%)	5379 (5%)	35 (8%)	3	Constructions
Real estate	6251 (11%)	1054 (1%)	3 (0%)	0	Real estate companies
Tech services	3505 (6%)	2318 (2%)	14 (3%)	4	Professional, scientific, technical activities
Hospitality	3074 (5%)	1101 (1%)	21 (4%)	7	Hotels and restaurants
Business support services	2197 (4%)	1611 (1%)	26 (6%)	12	Business support services, renting
Comm. and info serv.	2085 (3%)	1027 (1%)	10 (2%)	5	Communication and information services
Logistics	1594 (2%)	3102 (3%)	22 (5%)	13	Transport and warehousing
Agricolture	1403 (2%)	1727 (1%)	9 (2%)	6	Agriculture, farming and fishing
Healthcare	1077 (2%)	1070 (1%)	23 (5%)	21	Healthcare activities
Finance	718 (1%)	3222 (3%)	13 (3%)	19	Finance and Insurance activities
Utilities	708 (1%)	1210 (1%)	1 (0%)	2	Utilities
Education	466 (0%)	155 (0%)	2 (0%)	5	Education
Entertainment	445 (0%)	106 (0%)	2 (0%)	4	Arts and entertainment
Waste MGMT	434 (0%)	2045 (2%)	11 (2%)	26	Waste management services
Other serv	217 (0%)	65 (0%)	1 (0%)	5	Other service activities
Mining	153 (0%)	249 (0%)	1 (0%)	8	Mining companies
Public admin	4 (0%)	0 (0%)	0 (0%)	0	Public administration
Extra	3 (0%)	0 (0%)	0 (0%)	0	Foreign organizations

In particular, we used this data to compute the industrial composition of each municipality. We considered all the companies active during the earthquake (August 2016). We grouped companies accordingly to their industrial sector as specified by standard ATECO 1-Letter codes (e.g., Agriculture, Arts and Entertainment, Buildings, Commerce). For each group, we computed the total revenue. Finally, we normalized the count to sum to 1. The result represents the industrial composition of a given municipality (e.g., Municipality A has: Agriculture=0.8, Arts and Entertainment = 0, Construction = 0.2). We used the total revenue as a proxy of the relative weight of the company. We run experiments using the total number of employees, obtaining similar results.

#### Damage and recovery data

Governmental agencies in charge of designing policies for the reconstruction have devised a multi-step approach to collect data about damage and requests for recovery. Reporting these phases is essential to understand the nature of the data.

The Italian Civil Protection, the first responding agency to access the affected area during and after the population’s evacuation, initiated the damage assessment (first phase). The experts of Civil Protection had to fill in a specific administrative file in which they reported whether it was safe to access the building and the level of damage. If the building was unsafe (in Italian *inagibile*), the owners could request governmental funds to cover the rebuilding costs. These technical reports provide a first assessment of the damage, and we used them to compute the potential forecast amount of funding requests for the reconstruction. We called them RCR Forecast (*Richiesta Contributo per la Ricostruzione*). These files helped to develop a preliminary assessment of the amount of funding needed for the reconstruction, but this first step must be followed by several others. Only after a second request compiled by another expert in compliance with all the regulations has been submitted by the owner of the damaged building (residential, commercial, or industrial) do the governmental agency and the municipality allocate the necessary funds for reconstruction. Once the expert has submitted the request, the administration evaluates the report and responds. The number of requests for funding presented after these stages is what we define RCR Submitted. It is worth stressing that RCR Forecast and RCR Submitted are not identical. Therefore the two datasets may differ. This difference is relevant because the initial forecast is based only on the assessed damage of a given building, regardless of the owners’ decision to rebuild them. If the damage is considered negligible, homeowners may decide not to proceed with the request for funding because the procedure can be expensive and the fund received are insufficient to fix or renovate the damaged house. In addition, the damage assessment process can take between a few months and some years. In the meantime, individuals can decide to relocate and never come back. In this case, there is no willingness to continue the process to obtain funding, and a discrepancy between the initial damage assessment and the final one might emerge.

https://sisma2016data.it is the open data initiative (supported by the Italian Government) to monitor damage assessment and recovery plans for the earthquake in central Italy in 2016-2017. This dataset provides information about damage assessment and reconstruction, including the expected requests for rebuilding (RCR Forecast) and the actual ones presented by inhabitants (RCR Submitted) for each municipality. RCR Submitted is available both for private and public buildings in terms of absolute numbers of requests and total amount in euros.

1. **RCR Forecast**. The forecast number of requested funding (RCR) is calculated based on experts’ first assessment of the damage.
2. **RCR Submitted** The number of requests presented, which will be able to access the funding (although not necessarily accepted at the current date). These RCRs can be private (private buildings, including commercial activities) and public (including buildings belonging to the state, like schools, and religious buildings, such as churches). For each category, available data are the absolute number and the requested amount in Euros.

All the measures are normalized based on the number of inhabitants of the relative municipality.

#### Census and demographic data

We used data from the official census data about the resident population and changes in residency in the municipalities under study. Thanks to the census data estimated by ISTAT between 2002 and 2018, we deployed the municipal demographic balance. We extracted the number of residents and the number of residency changes in 2015, 2016 and 2017 from the database (https://demo.istat.it/ricostruzione/). The database allows the extraction of municipalities for each province and each year. Therefore we aggregated data at the yearly and provincial levels to obtain the entire dataset on population variation.

### Resident population from mobile phone

Several studies identified algorithms to extract resident population from mobile phone data^55–57^. The main criteria to consider is the average number of mobile phones at night during weekdays as a proxy for the resident population. We applied this procedure also in this work considering mobile phone data from 1 am to 5 am as “night” data. The resulting estimate is naturally lower than census data as we monitor just one telecom operator, and there might be bias in the sample population (e.g., children who do not have phones). We partially compensate for those biases by scaling our results by the telecom operator market share.

**Figure 7:**
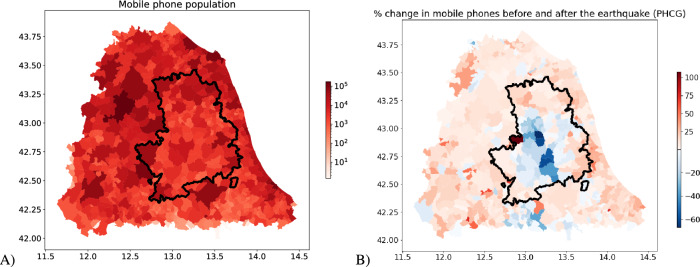
(**A**) Number of mobile phones for each municipality before the earthquake. (**B**) Percentage change of mobile phones before and after the earthquake (PCHG). Maps have been drawn using python libraries: *matplotlib 3.5.2*, *geopandas 0.2.12* – https://www.anaconda.com.

Figure 1A, presented above, shows the relationship between the resident population monitored by mobile phone data and actual census counts. Figure 7A shows the number of mobile phones for each municipality before the earthquake. Figure 7B presents the percentage change in mobile phones before and after the earthquake (PCHG).

### Forecasting mobile phone data

We created a forecasting model to predict population dynamics measured by mobile phones. The model trained on individual municipalities and macro-regions (crater and out-of-crater regions) with data before the first earthquake hit the area (24 August 2016). Then we used the model to forecast population dynamics.

The main idea of the adopted forecasting approach is to use non-autoregressive models. In this way, the model never receives information about the earthquake hit. Therefore – although we are fully aware that there might be several confounding factors and this is not a complete causal analysis – we think of the forecast as a counterfactual scenario in which the earthquake did not happen.

The forecasting model uses only features associated with the timestamp: year (*Y*), week of the year (*WoY*), days of the week (*DoW*), and hour (*H*). Features other than a year are considered categorical (and therefore one-hot encoded). The model uses these features to forecast the number of people (cell phones) in the area. We tested both a linear model and a decision tree regressor.

For example, in the linear model, for each municipality and macro-region, we fit a model like the one in the equation below. The training procedure learn parameters $\alpha$, $\beta _i$, $\gamma _j$, $\delta _k$ using training data before the earthquake. Then, it uses the equation to forecast future time steps. The case of the decision tree regressor is perfectly analogous.

$$\begin{aligned} \text {forecast}_t = \alpha * Y + \sum _{i=1}^{weeks} \beta _i * WoY_i + \sum _{j=1}^{7} \gamma _j * DoW_j + \sum _{k=1}^{24} \delta _k * H_k \end{aligned}$$

**Figure 8:**
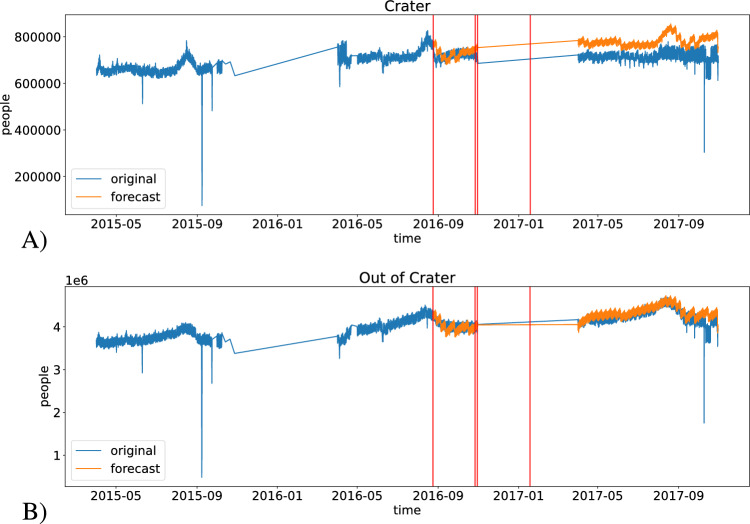
Forecast number of phones. (**A**) In crater regions. (**B**) Out of crater regions.

Figure 8 shows the forecast result -linear model - applied to all the regions in and out of the crater area. These in the crater area see an average decline in the mobile phone (MPE) of -6.5%. These out of the crater see an MPE of -1.5%. Results from decision tree regressor exhibit a similar pattern.

### Regression models

We developed several Ordinary Least Squares (OLS) models in our study. All the models try to explain MPE based on multiple data:

- We considered the industrial composition of each ATECO 1-Letter code (e.g., Agriculture, Arts and Entertainment, Buildings, Commerce) and the percentage of companies of that code. To limit the number of variables, we consider only those sectors contributing to at least 1% of the number of companies and 1% of the revenue of the area. Other sectors are discarded in Section “Results”. Nevertheless, we report the OLS with all the sectors in this Section.
- We computed the distance from the epicentre as the length from the closest epicentre of the earthquake.
- We considered the median altitude of the municipality as related to the municipality isolation.
- We considered information about damage assessment and reconstruction, including the expected requests for rebuilding (RCR Forecast) and the number of the actual request presented by inhabitants (RCR Submitted) for each municipality. RCR Submitted is available both for private and public buildings in terms of absolute numbers of requests and the total amount in euros.

In Section “Results”, we discussed the main findings of our model. In this Section, we show results analogous to Table 1, but using RCR forecast as a measure of damage assessment (see Table 5). The conclusions stated in Section “Results” hold in this case: RCR forecast contributes significantly and negatively to MPE, the more RCR (i.e., destruction) more the population displacement.

**Table 5:** Model to explain MPE (MPE INIT, MPE END, MPE) in terms of geographic and forecast damage features.

	MPE INIT	MPE END	MPE
D. from epicenter	0.292***	0.436***	0.402***
	(0.081)	(0.159)	(0.126)
ALT MED	$-$0.001	0.001	0.000
	(0.003)	(0.005)	(0.004)
RCR forecast	$-$6.090***	$-$18.244***	$-$15.339***
	(1.747)	(3.407)	(2.711)
Observations	122	122	122
$R^2$	0.475	0.512	0.558
Adjusted $R^2$	0.461	0.500	0.547
Residual Std. error	6.706	13.079	10.410
F statistic	35.534***	41.304***	49.741***

*p < 0.1; **p < 0.05; ***p < 0.01.

Similarly, we report results using all the ATECO codes. The introduction of the “minor” sectors (i.e., public administration, entertainment, mining, waste management, extraterritorial organization, education, and other services) does not affect the results significantly (see Table 6).

**Table 6:** Model to explain MPE (MPE INIT, MPE END, MPE) in terms of geographic, damage and economic features. All the ATECO sectors are considered.

	MPE INIT	MPE END	MPE
Distance from epicenter	0.265***	0.564***	0.492***
	(0.087)	(0.176)	(0.140)
ALT MED	0.002	$-$0.005	$-$0.003
	(0.003)	(0.006)	(0.005)
RCR PRIV	$-$21.315*	$-$10.550	$-$13.123
	(11.915)	(24.000)	(19.105)
RCR PUB	$-$165.588***	$-$413.376***	$-$354.161***
	(53.468)	(107.695)	(85.732)
Agricolture	$-$6.563	$-$10.076	$-$9.237
	(4.816)	(9.701)	(7.723)
Real estate	0.411	$-$20.000	$-$15.122
	(8.887)	(17.901)	(14.250)
Hospitality	$-$15.972**	$-$9.690	$-$11.191
	(6.114)	(12.315)	(9.803)
Manufacturing	$-$8.780**	$-$22.293***	$-$19.064***
	(3.474)	(6.997)	(5.570)
Tech services	$-$9.865	$-$32.338	$-$26.967
	(10.888)	(21.931)	(17.458)
Whole/retail sales	$-$8.931**	$-$20.960**	$-$18.085***
	(3.986)	(8.029)	(6.391)
Constructions	$-$13.752***	$-$13.564*	$-$13.609**
	(3.918)	(7.891)	(6.281)
Business support services	$-$10.799	$-$22.865	$-$19.981
	(21.446)	(43.196)	(34.387)
Communication and info serv.	5.754	$-$59.279	$-$43.738
	(47.455)	(95.583)	(76.090)
Logistics	$-$11.688	$-$38.591*	$-$32.162**
	(9.902)	(19.945)	(15.877)
Finance	$-$6.289	$-$44.935***	$-$35.700***
	(7.012)	(14.123)	(11.243)
Utilities	$-$11.470	$-$38.441*	$-$31.996*
	(10.276)	(20.698)	(16.477)
Public admin	0.000	0.000	0.000
	(0.000)	(0.000)	(0.000)
Entertainment	$-$82.666	99.584	56.030
	(128.636)	(259.099)	(206.259)
Mining	1.006	$-$18.761	$-$14.037
	(79.348)	(159.822)	(127.229)
Waste MGMT	$-$14.306	8.356	2.940
	(11.811)	(23.790)	(18.938)
Extra	0.000	0.000	0.000
	(0.000)	(0.000)	(0.000)
Healthcare	$-$35.299	$-$56.924	$-$51.756
	(32.295)	(65.049)	(51.783)
Education	113.221	$-$51.497	$-$12.133
	(93.092)	(187.505)	(149.267)
Other serv	1057.701	627.047	729.963
	(1489.736)	(3000.615)	(2388.686)
Observations	123	123	123
$R^2$	0.557	0.572	0.610
Adjusted $R^2$	0.460	0.478	0.525
F statistic	5.717***	6.073***	7.120***

*p < 0.1; **p < 0.05; ***p < 0.01.

### Spatial auto-correlation

**Figure 9:**
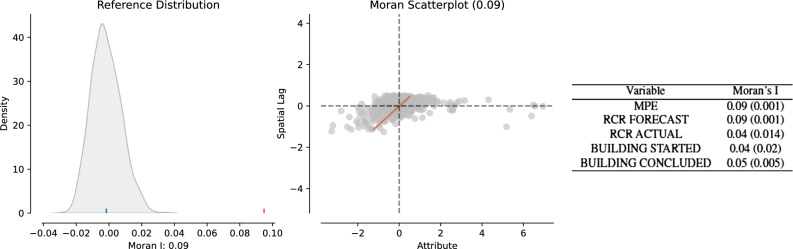
Spatial auto-correlation analysis. Moran’s plot for MPE. In the table Moran’s I for all the main variables.

We tested the spatial auto-correlation for all the main variables involved. The presence of spatial auto-correlation is trivial given the localized nature of the earthquake, as it is also apparent in Fig. 3C. Figure 9 presents Moran’s analysis of all the main considered variables. The presence of spatial auto-correlation hints at the influence of the earthquake on the monitored variable.

### Predict variation

In this section, we propose an approach complementary to the one presented in the above results to analyze MPE after the earthquake. The idea is to create a set of variables summarizing (and reducing the dimensionality of) the features describing different municipalities. Then, we apply a clustering mechanism to the resulting factors.

We consider and transform the variables relevant to the population variation in the earthquake areas.

The considered features are calculated for each municipality as follows:

- The number of companies in the construction sector (*Constructions*);
- The number of companies in the accommodation and restaurant sector (*Hospitality*);
- The number of companies in the wholesale and retail trade sector (*Whole/retail sales*);
- The number of companies in the professional services sector (*Tech services*);
- The number of companies in the renting sector (*Business support services*);
- The total number of employees (*Employees*);
- The mean altitude in meters (*Alt med*);
- The province (*Province*);
- The municipality (*Municipality*);

Factor analysis has enabled us to transform and reduce the number of selected features. Initially, we considered a higher number of features. The final selection has been performed based on trial and error and according to the proportion of each variable’s variance explained by the factors (commonalities).

**Figure 10:**
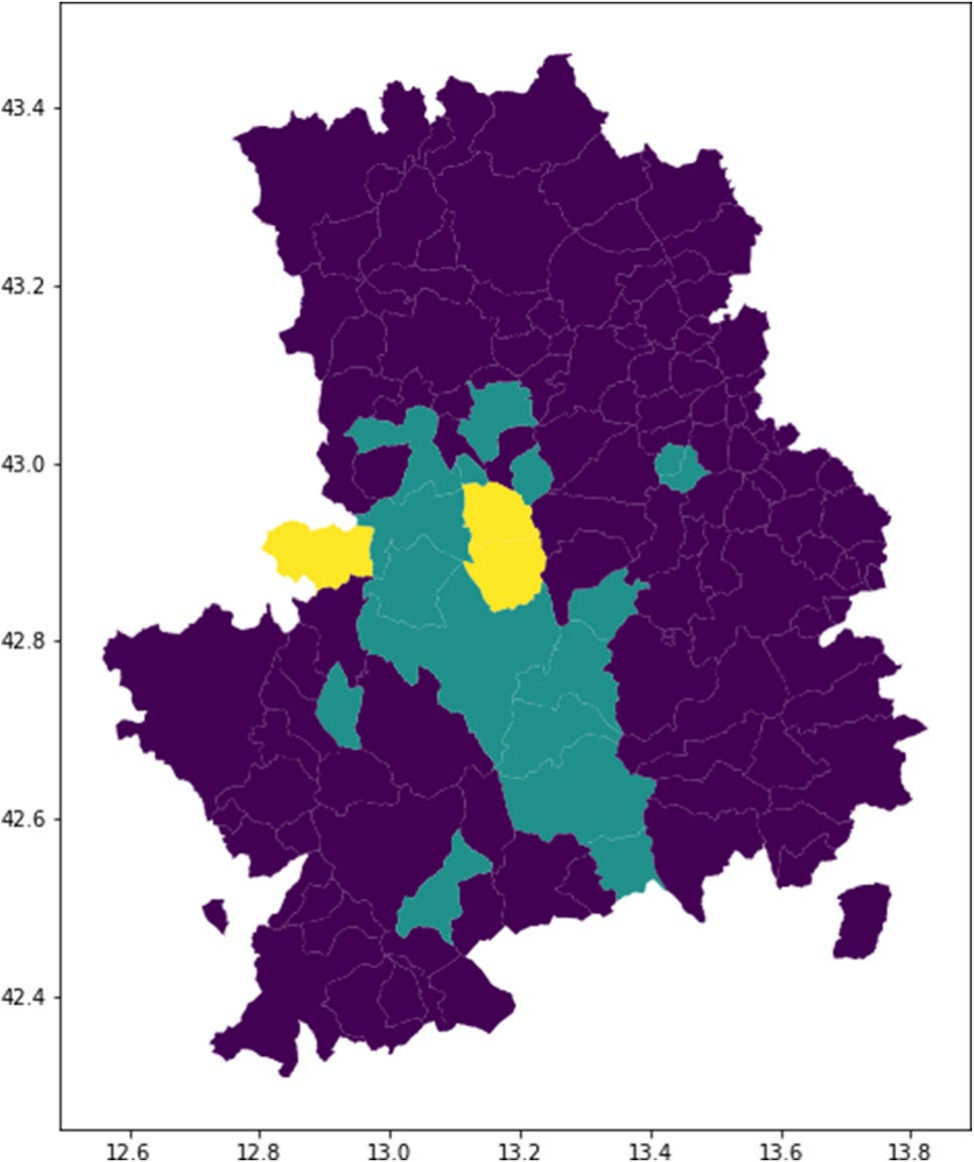
**(left)** Results of the factor analysis using a Varimax rotation of the axes. **(right)** Map representing the three clusters of municipalities created based on the new factors. Map has been drawn using python libraries: *matplotlib 3.5.2*, *geopandas 0.2.12* – https://www.anaconda.com.

We applied Kaiser Varimax rotation^58^ on the above set of variables to identify factors that synthesize the considered variables. Based on the results, we identified three factors representing the variables. Figure 10 presents the results: Factor 1 has high factor loadings for *Constructions*, *Hospitality*, *Whole/retail sales*, *Tech services*, *Business support services* and *Employees*. Therefore, we named this factor as **Industrial_structure**. The second factor formed using the variable *Alt_med* was named **Physical_characteristics**. Finally, the third factor is the result of *Province* and *Municipality* so that it has been named **Administrative_characteristics**. The commonalities of each variable are above 77%. Therefore it is possible to conclude that the identified factors reasonably and reliably explain the observed variables. We applied the factors to the entire dataset and created three new variables, summarizing the above features.

Based on the three newly created factors, we implemented an agglomerative cluster analysis to identify three clusters of municipalities. Figure 10 shows that one group of municipalities is concentrated in the crater area even though we did not deploy the distance from the epicentre to create the clusters. Furthermore, three have a substantial variation in the number of mobile phones. The similarity of this map with Figs. 3C and 7B confirm the impact of industrial, physical and administrative features to explain and predict mobile phone variation (MPE).

## Data Availability

Data and code to reproduce our experiments is available at https://github.com/mmamei/earthquake2016.
